# Occurrence of Adenovirus in Fecal Samples of Wild Felids (*Panthera onca* and *Leopardus pardalis*) from Brazil: Predators as Dispersing Agents?

**DOI:** 10.3390/vetsci11100511

**Published:** 2024-10-17

**Authors:** Ygor Machado, Laís Santos Rizotto, Hilton Entringer Jr., Helena Lage Ferreira, Gabriel Augusto Marques Rossi, Ana Carolina Srbek-Araujo

**Affiliations:** 1 Programa de Pós-Graduação em Ciência Animal, Universidade Vila Velha, PPGCA-UVV, Vila Velha 29102-920, ES, Brazil; ygo.machado@gmail.com (Y.M.); gabriel.rossi@uvv.br (G.A.M.R.); 2 Programa de Pós-Graduação em Epidemiologia e Saúde Única, Departamento de Medicina Veterinária Preventiva e Saúde Animal, Faculdade de Medicina Veterinária e Zootecnia, Universidade de São Paulo, FMVZ-USP, São Paulo 05508-270, SP, Brazil; laisrizotto@gmail.com (L.S.R.); hlage@usp.br (H.L.F.); 3 Centro Para el Estudio de Sistemas Marinos, Centro Nacional Patagónico (CCT-CONICET), and Facultad de Ciencias Naturales y Ciencias de la Salud, Universidad Nacional de la Patagonia San Juan Bosco (UNPSJB), Puerto Madryn U9120ACD, Argentina; hiltonentringer@hotmail.com; 4 Departamento de Medicina Veterinária, Faculdade de Zootecnia e Engenharia de Alimentos, Universidade de São Paulo, FZEA-USP, Pirassununga 13635-000, SP, Brazil

**Keywords:** *Leopardus pardalis*, Mastadenovirus, *Panthera onca*, protected area, viruses

## Abstract

Wild felids are vital to maintaining the ecological balance in natural environments as they regulate prey populations at different levels of the food chain. Changes in the dynamics of predator populations can impact the entire biodiversity of an ecosystem. There are few reports of Adenovirus infections in these animals, and little is known about their epidemiology. Therefore, a deeper understanding of these viruses within a One Health framework is essential, given their importance to animal, human, and environmental health. This study aimed to detect Adenovirus DNA in fecal samples of wild felids from a remnant of the Atlantic Forest in southeastern Brazil, renowned for its high biodiversity. A total of 43 fecal samples, 11 from jaguar (*Panthera onca*) and 32 from ocelot (*Leopardus pardalis*), were collected. The samples were subjected to viral nucleic acid extraction and genetic material amplification through PCR, followed by nucleotide sequencing. All phylogenetic analyses were based on the amino acid sequences of the DNA polymerase and IV2a genes. Adenovirus DNA was detected in the feces of both species, with two samples of each feline testing positive. This study reports, for the first time, the occurrence of Adenovirus associated with feces of *Panthera onca* and *Leopardus pardalis*. All detected sequences were grouped within the *Mastadenovirus* genus. Based solely on phylogenetic distance criteria, the identified sequences could be classified as *Mastadenovirus bosprimum* and *Mastadenovirus* from the vampire bat *Desmodus rotundus*. We hypothesize that Adenoviruses were associated with the prey consumed, which may allow the felines to act as eventual viral dispersing agents in the environment, in addition to the risk of being infected. This study provides new information on the association of Adenoviruses with wild felids and their prey, and offers important insights into the ecological dynamics of these viruses in natural environments. It suggests that wild felines may play a crucial role in viral surveillance programs.

## Simple Summary

Wild felids play a crucial role in maintaining the ecological balance within natural environments, which are increasingly affected by anthropogenic activities. Various viruses can infect these animals, and there are only two documented cases of Adenovirus occurrence in wild felids. This study aimed to detect Adenovirus DNA in fecal samples of wild felids from a remnant of the Atlantic Forest in southeastern Brazil. Adenovirus DNA presence was identified in four distinct fecal samples from jaguar (*Panthera onca*) and ocelot (*Leopardus pardalis*). This is the first report of this virus associated with *Panthera onca*. The viral DNA sequences were classified within the genus *Mastadenovirus*, specifically related to *Mastadenovirus bosprimum* and *Mastadenovirus* from the vampire bat *Desmodus rotundus*. We hypothesized that Adenoviruses were associated with the prey consumed, which may allow the felines to act as eventual viral dispersing agents, in addition to the risk of being infected. This study contributes to a better understanding of the occurrence and epidemiology of Adenoviruses in wild felids and their prey. A deeper understanding of these viruses within a One Health framework is crucial, given their relevance to animal health, human health, and environmental conservation.

## 1. Introduction

The decline in biodiversity, increased trade and global transportation, rapid urbanization, climate change, and other anthropogenic factors contribute to the rise and spread of viral pandemics [1,2]. In this sense, maintaining biodiverse and healthy ecosystems may play a crucial role in safeguarding humans and wildlife against the emergence and transmission of infectious diseases [3,4]. In a scenario that emphasizes the One Health concept, where human, animal, and environmental health are inseparable, it is crucial to understand the occurrence and the ecological dynamics of viruses in natural environments. Brazil is one of the countries with the greatest biodiversity, with an estimated occurrence of 9.5% of all known species on Earth, totaling between 170,000 and 210,000 species [5]. However, Brazilian biodiversity is threatened by human activities that have resulted in the loss of a large part of the natural habitats in highly biodiverse biomes (e.g., Atlantic Forest [6] and Cerrado [7]). Therefore, it must be considered that the weakening of Brazilian natural ecosystems may contribute to the emergence of diseases.

Among all existing biodiversity, the Felidae family is particularly notable, as it includes only species recognized as predators, including small, meso, and top predators. These species are essential for maintaining the ecological balance by regulating the populations of other predators and prey species [8,9]. The consumption of sick and more vulnerable prey can be expected, contributing to suppressing or limiting disease prevalence (e.g., [10]). In this sense, in a competitive context, the inclusion (exotic species) or exclusion of predators could influence disease dynamics [11]. Consequently, changes in the community composition or abundance of wild felids can significantly disrupt the equilibrium of an ecosystem (e.g., [12]). This highlights that the conservation of predators can have direct implications for promoting One Health since these animals themselves can contribute to the maintenance of biodiversity and provide other ecosystem services related to disease control.

On the other hand, when interacting with infected prey, predators may also become infected [13] and disseminate diseases in the environment during slaughter, consumption, and deposition of feces containing the remains of infected prey, effectively acting as dispersing agents [14]. Infections, including those caused by viruses, can significantly affect the populations of wild felines and their interactions with the prey and the environment [15]. Therefore, different diseases could represent threats to Brazilian felines, highlighting that, of the nine currently recognized species of wild felids in the country [pampas cat (*Leopardus colocolo*), Geoffroy’s cat (*Leopardus geoffroyi*), ocelot (*Leopardus pardalis*), northern tiger cat (*Leopardus tigrinus*), southern tiger cat (*Leopardus guttulus*), margay (*Leopardus wiedii*), jaguarondi (*Herpailurus yagouaroundi*), puma (*Puma concolor*), and jaguar (*Panthera onca*) [16], 88.9% are experiencing a global trend of population decline and 55.6% are classified in some threat category [17]. Recently, a new species of Adenovirus was detected in a wild *Leopardus pardalis* in Brazil [18]. The animal, which had been hit by a vehicle, was examined using molecular biology techniques, revealing the presence of “ocelot adenovirus 1” causing a systemic infection [18]. Before this study, the presence of Adenovirus in wild felids had been documented only once worldwide in a captive leopard (*Panthera pardus*) in India [19].

Adenoviruses are non-enveloped viruses with an icosahedral shape, measuring around 70–90 nm in diameter, and contain a linear double-stranded DNA genome that ranges from 24 to 48 kb. These viruses belong to the Adenoviridae family, divided into six genera. Among them, *Mastadenovirus* is the only genus that infects mammals [20,21,22], which includes over 50 species [21]. The other genera are *Aviadenovirus*, comprising 14 species that infect birds; *Barthadenovirus*, with 9 species affecting reptiles, birds, ruminants, and marsupials; *Siadenovirus*, including more than 7 species that infect birds, frogs, and tortoises; *Ichtadenovirus*, represented by a single species found in white sturgeon; and *Testadenovirus*, with one species affecting red-eared sliders [21]. Adenoviruses can cause persistent infections [18] and can be transmitted through direct contact and indirectly via saliva, respiratory secretions, feces, or urine. Adenoviruses are generally species-specific, although there is evidence of zoonotic transmission between humans and non-human species, including primates, bats, felines, swine, canines, sheep, and goats. These viruses are believed to have crossed, and will likely continue to cross, host species barriers [23].

Therefore, a better understanding of the Adenoviruses capable of infecting wild animals is needed within a One Health approach, given their significance for animal health, including that of wild felids, as well as human health and environmental balance. In this context, the present study reports the molecular detection and description of Adenovirus in fecal samples of wild felids from a remnant of the Atlantic Forest in southeastern Brazil.

## 2. Materials and Methods

### 2.1. Study Area

**Figure 1:**
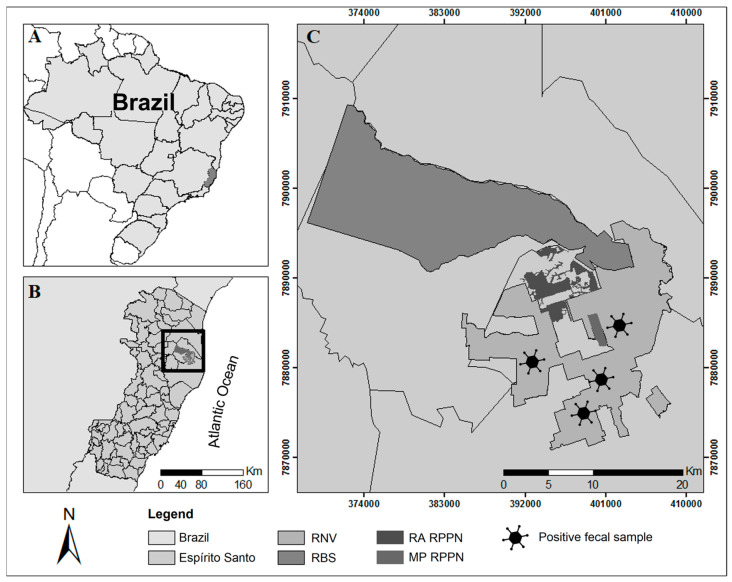
Linhares-Sooretama Block, located in the northern region of Espírito Santo, southeastern Brazil (**A**,**B**), with a detailed view of the Vale Natural Reserve (RNV), Sooretama Biological Reserve (RBS), and the Private Natural Heritage Reserves of Recanto das Antas (RA RPPN) and Mutum Preto (MP RPPN) (**C**). Geographic coordinates: Universal Transverse Mercator (UTM Zone 24K).

Data collection was carried out in the Vale Natural Reserve (Reserva Natural Vale—RNV) (19°01′16″ S–19°14′49″ S and 40°05′22″ W–39°52′06″ W). This privately protected area is located in the northern region of Espírito Santo, between the municipalities of Linhares and Jaguaré, Brazil, covering approximately 22,711 hectares. The RNV, together with the Sooretama Biological Reserve (Reserva Biológica de Sooretama—RBS), the Recanto das Antas Private Natural Heritage Reserve (Reserva Particular do Patrimômio Natural—RPPN), and the Mutum Preto RPPN, forms the Linhares-Sooretama Block (Bloco Linhares-Sooretama—BLS), which represents a continuous forest area of about 53,000 hectares. The BLS is the largest continuous remnant of native vegetation in Espírito Santo. It represents one of the largest remnants of the lowland Atlantic Forest in Brazil, recognized as an important conservation area for large mammals [24]. For details regarding the location and context of the study area, please view Figure 1 in the Results section.

The vegetation of the RNV consists of various types, including dense forests, muçununga forests, flooded swamps, and native grasslands [25]. The dense forest, or the “tabuleiro” forest, is the predominant formation and is classified as a Perennial Seasonal Forest [26]. The climate in the region is tropical Fwith a dry winter [27], with an average annual temperature of 24.3 °C ± 2.1 and an average precipitation of 1214.6 mm ± 260.6 [26]. The RNV contains an internal network of unpaved roads of approximately 126 km long, which provide access to all areas and phytophysiognomies of the reserve. The surroundings of the RNV are primarily composed of agricultural activities, such as livestock farming and the cultivation of papaya, coffee, and eucalyptus [26].

### 2.2. Fecal Sample Collection and Feline Identification

This study analyzed fecal samples collected between May 2017 and December 2018. The samples were obtained through active searches during linear transects conducted by walking along the internal roads of the RNV, as well as on the embankments and firebreaks bordering neighboring properties. The transects were carried out during monthly campaigns, each lasting four days. In each campaign, sampling was performed to cover the largest possible area, covering a total distance of 1668 km. The collected samples were stored in plastic bags and labeled with an individual registration code for subsequent analysis and identification of the species in the laboratory. Only fresh fecal samples were sent for viral analysis. A fraction of each original sample was separated in the study area and stored in sterile Falcon tubes. The samples were kept refrigerated at temperatures between 2 and 8 °C in the field (cooler with ice) and frozen at −20 °C in the laboratory until processing.

The identification of the species that deposited the fecal samples was conducted through microstructural analysis (cuticle and medulla) of guard hairs ingested by predators during self-grooming or prey consumption [28]. The same procedure supplemented by the morphological analysis of other undigested items (such as teeth, bones, feathers, and scales) was applied to identify the prey species [29].

### 2.3. Suspension of Fecal Samples, DNA Extraction and Amplification

Approximately 200 mg of each fecal sample were used, diluted to a 20% (*w*/*v*) concentration in 1000 μL of Tris-Calcium buffer (Tris 0.01 M, CaCl_2_ 1.5 mM, pH 7.2). The samples were homogenized using a vortex mixer (Biomixer, Araraquara, Brazil) and then centrifuged in a refrigerated microcentrifuge (Nova Tecnica Ltd.a., Piracicaba, Brazil) at 3000 rpm for 10 min. After transferring 700 μL of the supernatant, the resulting dilution was stored at −20 °C until viral nucleic acid extraction.

The steps for extracting and amplifying viral genetic material were carried out following the protocol established by Wellehan and colleagues [30], using a nested-PCR targeting the DNA polymerase gene. For the first amplification, the following primers were used: forward primer (TNMGNGGNGGNMGNTGYTAYCC) and reverse primer (GTDGCRAANSHNCCRTABARNGMRTT); for the second reaction, forward primer (CANCCBCDRTTRTGNARNGTRA) and reverse primer (GTNTWYGAYATHTGYGGHATGTAYGC) were used. Respectively, these primers produced fragments of approximately 550 and 318–324 bp. Additionally, for the amplification of the IVa2 gene, the forward (CCNNSNCCNGARACNGTNTTYTT) and reverse (GGRTTCATRTTRTGNARNACNAC) primers were used for the first reaction, which generate products of 397 bp, as well as the following primers (CCNCARRTNGAYATGATHCCNCC) and (TTNSWNGGRAANGCRTGRAARAAYTT), for the second reaction, which generate products of 302 bp [31].

The reaction volume was 50 µL, consisting of 37 µL of ultrapure sterilized water, 5 µL of 10× REDTaq DNA polymerase buffer (Sigma-Aldrich, St. Louis, MO, USA), 1 µL of MgCl_2_ solution (25 mM), 1.5 µL of dNTP mix (10 mM), 1 µL of each primer (50 µM), 2.5 µL of REDTaq DNA polymerase enzyme (Sigma-Aldrich, St. Louis, MO, USA), and 1 µL of sample (material obtained from the suspension step). The DNA fragments were purified using the NucleoSpin II kit (Macherey-Nagel, Düren, Germany). PCR products, showing a single band of the correct size containing only a specific product, were purified using the ExoProStar 1-Step kit (Cytiva, Marlborough, MA, USA); otherwise, their specific band was gel purified using the NucleoSpin Gel and PCR Clean-up kit (Macherey-Nagel, Düren, Germany). Purified PCR products were Sanger sequenced.

### 2.4. DNA Sequencing and Phylogenetic Analyses

The direct sequencing of the amplified and purified products was performed using appropriate internal primers in a 10 µL reaction volume with the commercial “Big Dye Terminator^®^ v1.3 Cycle Sequencing Kit” (Applied Biosystems, Carlsbad, CA, USA), according to the manufacturer’s instructions. Electrophoresis was conducted using the ABI PRISM 3100 machine (Applied Biosystems, Carlsbad, CA, USA).

The sequences were analyzed and manually edited using the Chromas software v2.6.6 (Technelysium, Queensland, Australia), and then verified using the Basic Local Alignment Search Tool (BLASTn) to confirm their identity. Subsequently, the nucleotide sequences were translated into amino acid sequences. Their open reading frame was confirmed using the Expasy platform [32], and amino acid identities were verified using the Basic Local Alignment Search Tool (BLASTp). The sequences obtained in this study have been deposited in GenBank (accession numbers PQ347813 to PQ347817).

All phylogenetic analyses were based on the amino acid sequences of the DNA polymerase and IV2a genes separately. For each gene, the sequences obtained were aligned using ClustalW in the MEGA7 software [33] with representative sequences of all Adenovirus genera available in GenBank (Table S1). The most appropriate evolutionary model for the analyses and the identity matrices were estimated using MEGA7. Phylogenetic trees were constructed using the Maximum Likelihood method with the Le Gascuel model, a gamma distribution (LG+G), and 1000 bootstrap replicates, utilizing MEGA7. The trees were then edited using FigTree software v1.4.4.

## 3. Results

A total of 43 fecal samples were collected and processed for viral nucleic acid extraction and amplification. Of these, 11 samples (25.6%) were from *Panthera onca*, and 32 samples (74.4%) were from *Leopardus pardalis*. Adenovirus DNA was detected in four of these samples, with two positive samples from each species. The positive fecal samples were collected on different dates: VS631 in June 2017, VS649 in December 2017, VS652 in April 2018, and VS655 in January 2018. The sampling locations varied in distance (Figure 1), ranging from 4.08 km to 10.21 km, with an average distance of 7.65 km between them.

Analysis was performed on four partial sequences of the DNA polymerase coding gene (VS631 and VS649 from *Panthera onca*; VS652 and VS655 from *Leopardus pardalis*) and one partial sequence of the IVa2 gene (VS631 IVa2 from *Panthera onca*). All detected sequences clustered within the genus *Mastadenovirus*. The amino acid identities among the wild feline Adenovirus from Brazil, identified as Felid mastadenovirus/RK278 (MZ147500) [18], and the sequences obtained in our study were low, with percentages as follows: VS631 46.3%, VS649 53.8%, VS652 49.2%, and VS655 61.2%. This low level of identity suggests that the Adenovirus DNA detected in our study represents different species than the previously identified wild feline Adenovirus.

**Figure 2:**
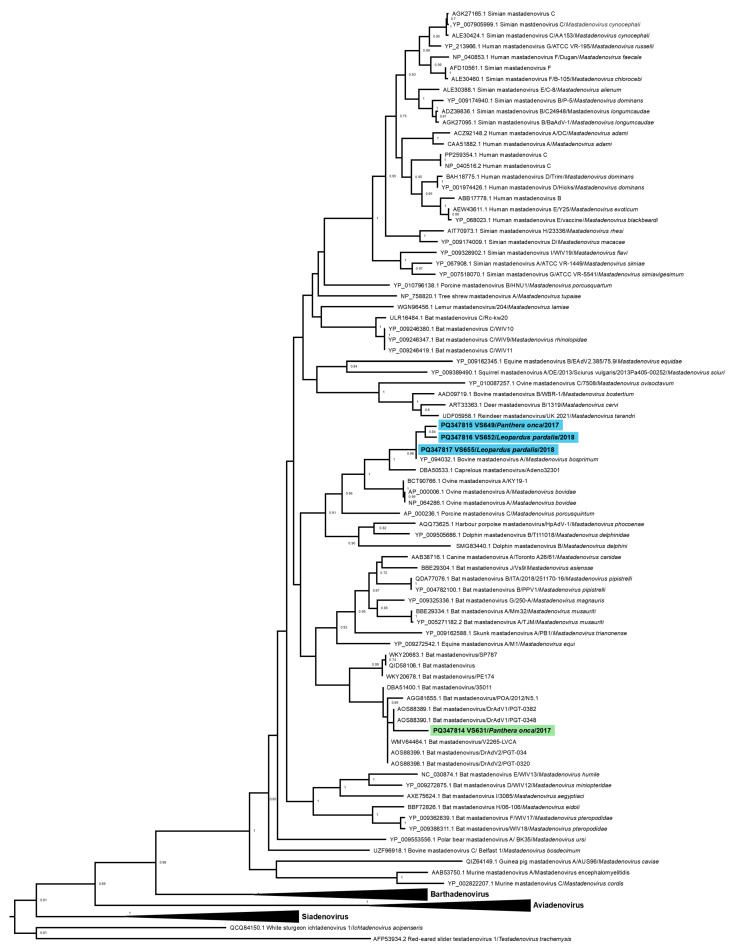
A phylogenetic tree based on amino acid sequences of Adenovirus DNA polymerase was generated using the Maximum Likelihood method with the Le Gascuel model and a discrete gamma distribution (LG+G), totalizing 905 positions in the final dataset. The analysis involved 103 amino acid sequences. Sequences detected in this study are highlighted and colored. Values lower than 70% were hidden.

The DNA polymerase gene sequences VS649, VS652, and VS655 clustered with *Mastadenovirus bosprimum* (YP_094032) (bovine adenovirus 1) and *Caprelous mastadenovirus* (DBA50533) [34]. According to the identity matrix, the obtained sequences have 92.6% and 100% identity with *Mastadenovirus bosprimum* sequences. Additionally, sample VS655 also showed 88.2% identity with the sequence of *Caprelous mastadenovirus* (Figure 2, Table 1).

The sequence VS631 clustered with mastadenoviruses from vampire bat *Desmodus rotundus* originating in Guatemala (AOS88389 and AOS88390) [35], showing 87% identity with these sequences (Figure 2, Table 1). The sample also had 83.3% identity with a mastadenovirus from *Desmodus rotundus* in Brazil (AGG81655), which has been suggested to be classified as Bat mastadenovirus T [22].

**Figure 3:**
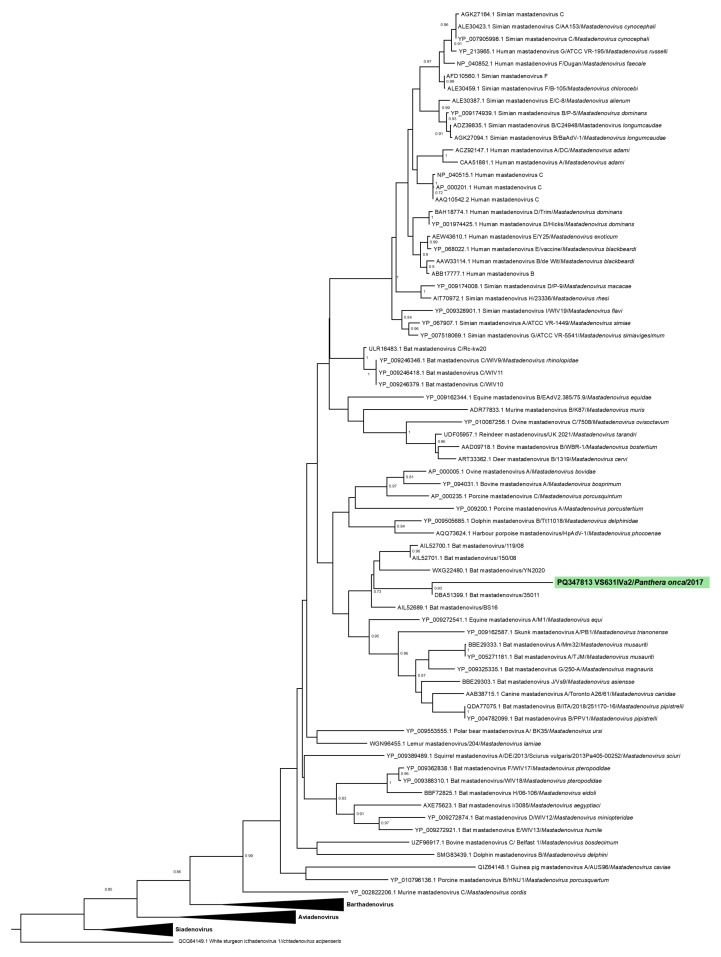
A phylogenetic tree based on amino acid sequences of Adenovirus IVa2 gene was generated using the Maximum Likelihood method with the Le Gascuel model and a discrete gamma distribution (LG+G), totaling 333 positions in the final dataset. The analysis involved 91 amino acid sequences. Sequences detected in this study are highlighted and colored. Values lower than 70% were hidden.

The sequence VS631 IVa2 from the IVa2 gene clustered with a mastadenovirus sequence from *Desmodus* sp. (DBA51399) [34], showing the highest identity of 68.55% with this sequence (Table 2, Figure 3).

According to ICTV, to determine the species of Adenovirus, at least two criteria had to be used: (1) a phylogenetic distance of greater than 10–15% based on the amino acid sequences of the DNA polymerase gene, and (2) other factors such as genomic organization (the E3 region), nucleotide composition, host range, oncogenicity in rodents, cross-neutralization, recombination capability, the number of virus-associated RNA (VA RNA) genes, and hemagglutination [21]. Based on phylogenetic distance alone, sequences VS649, VS652, and VS655 could be classified as *Mastadenovirus bosprimum*, while sequence VS631 would be associated with the same mastadenovirus species found in bats from Guatemala.

## 4. Discussion

Molecular analysis successfully detected the presence of Adenovirus DNA in four distinct fecal samples of wild felines in RNV. The interaction with these viruses was confirmed for two feline species (*Leopardus pardalis* and *Panthera onca*), also highlighting the association of Adenovirus with a meso and a top predator. However, it was not possible, without fecal DNA analyses, to determine whether it was associated with different individuals of the same species. Additionally, we emphasize that, in this study, the detection of viral DNA in the fecal samples was conducted, but the integrity of the viral particles, as well as their viability or infectious potential through viral isolation, were not assessed.

Adenovirus has been documented in both domestic [36] and wild felines [18,19]. In wild cats, the initial report described a case of anorexia, hepatitis with intranuclear inclusion bodies, myocarditis, renal lesions, and gastrointestinal tract lesions in a *Panthera pardus* death in a zoo in India [19]. More recently, a systemic Adenovirus infection was detected in a *Leopardus pardalis* roadkilled in Brazil. The virus was identified in samples from the brain, skeletal muscle, spleen, mesenteric lymph node, stomach, and duodenum, while samples from the kidney, cecum, lung, liver, and tongue were negative. The assessment of lesions was limited due to trauma and subsequent tissue damage. Ultimately, the Adenovirus was most similar to sequences previously described in pinnipeds from Peru and the United States. The authors proposed naming it “ocelot adenovirus 1” and suggested it may represent a new species within the *Mastadenovirus* genus [18]. The low similarity between this wild feline Adenovirus and the sequences from our study suggests that the Adenoviruses we identified belong to different species than the previously identified Adenovirus.

In our study, we report for the first time the occurrence of Adenovirus DNA in fecal samples from *Panthera onca* and *Leopardus pardalis*, and the first association with the jaguar independent of the biological material analyzed. For *Panthera onca* feces, one sequence (VS649) was identified as *Mastadenovirus bosprimum* (YP_094032), while the sequence VS631 clustered with mastadenovirus sequences from *Desmodus rotundus* originating in Guatemala (AOS88389 and AOS88390). To a lesser extent, the VS631 IVa2 sequence, from the IVa2 gene, clustered with a mastadenovirus sequence from *Desmodus* sp. (DBA51399). For the sequences obtained from fecal samples of *Leopardus pardalis*, all were grouped with *Mastadenovirus bosprimum* (YP_094032). Our findings further support the association of Adenovirus with *Leopardus pardalis* as recently reported [18].

Since the detection of Adenovirus genetic material was performed in fecal samples of wild felines, it cannot be determined whether the viruses were infecting these animals, as previously described [18], or were derived from prey and subsequently excreted along with their remains in the collected feces. The presence of viruses remaining in the feces of predators has been suggested in other studies involving small carnivores. This was attributed to viruses effectively present in predators [37], acquired via diet from infected prey [37,38] or related to the consumption of prey with infected intestinal parasites [38]. When evaluating the detectability period of rodent remains in the feces of *Leopardus pardalis* fed in captivity, it was observed that rodent hairs were most abundant on the second day, decreased significantly from the third day onward, and were no longer detectable after the fifth day post-consumption [39]. This may suggest that predators possibly disperse viral genetic material associated with prey in multiple events, and it may take days for all the virus’s material to be eliminated from the digestive system. Therefore, in addition to predators controlling diseases through predation, they can contribute to the spread of viruses in the environment, if these microorganisms are viable and infectious after passing through the predator’s digestive tract. The effective transmission of viruses (infectious forms) present in prey through fecal samples from predators has already been demonstrated experimentally [14].

In conjunction with this study, the diet of *Panthera onca* [29] and *Leopardus pardalis* (unpublished data) was evaluated, using the same fecal samples that underwent viral analysis. The dietary data indicate a high consumption of wild ungulates by *Panthera onca* in the RNV, with the samples showing evidence of peccaries (Tayassuidae), deer (Cervidae), and tapirs (Tapiridae) among the consumed prey. Other studies have identified anti-bovine Adenovirus antibodies in red deer (*Cervus elaphus*) and roe deer (*Capreolus capreolus*) in Great Britain [40], and in Yezo sika deer (*Cervus nippon yesoensis*) in Japan [41]. Additionally, a novel Adenovirus was isolated from a white-tailed deer (*Odocoileus virginianus*) in the United States, showing a 76% similarity to “Bovine Adenovirus Type 3” [42]. These findings suggest that close contact between wild cervids and domestic cattle may facilitate the transmission of viruses and their adaptation to new host species, raising concerns about managing both wild and domestic ungulates [42]. The analysis of fecal samples provides no evidence that *Panthera onca* has consumed domestic cattle in the RNV [29]. However, personal observations and camera trap records confirm the occasional presence of domestic cattle within the reserve, near the boundaries (unpublished data). Therefore, it is proposed that the detected viral sequences may have resulted from the infection of wild ungulates, with viruses possibly introduced by domestic animals in the surrounding areas. We also emphasize that the samples in this study were collected from areas further inland within the reserve, where domestic cattle are not present, ruling out the possibility of environmental contamination of fecal samples. Alternatively, the sequences might represent novel, previously undescribed viruses affecting wild ungulates, which are not currently included in the available databases.

The presence of *Mastadenovirus bosprimum* in the feces of *Leopardus pardalis* further supports the previously suggested hypothesis that the occurrence of this virus group is linked to the consumption of wild ungulates infected with the virus, as the predation of brocket deer (Cervidae) has been documented in the diet of this feline [43,44], including within the RNV [45]. However, no remains of domestic ungulates were observed in the *Leopardus pardalis* feces samples analyzed in the study area.

There is a great genetic diversity of Adenoviruses circulating in bats from the Brazilian Atlantic Forest [22]. However, there are no records of bat predation by *Panthera onca* in the study area [29] or other locations throughout its range [46]. Despite this, *Desmodus rotundus* preys on different species of mammals, such as tapirs (*Tapirus terrestris*) in BLS [47], one of the jaguar preys in the study area [29]. The risk of viral spillover from bats to other animal species, including humans, is well documented, either through direct or indirect contact [48,49,50]. Bat-origin viruses have been reported to infect horses with the Hendra virus [51], and humans with the Nipah virus [52], as well as transmit the rabies virus to various wild and domestic mammal species [53]. In laboratory settings, Adenoviruses isolated from bat feces have been shown to effectively infect human, monkey, dog, and pig cells [54]. As previously discussed for *Mastadenovirus bosprimum*, our findings suggest that the presence of a bat-related virus in our sample may not necessarily be due to bat predation, but rather to a viral infection affecting other species preyed upon by *Panthera onca* in RNV. Moreover, it is likely that this prey was infected by an Adenovirus that underwent spillover, probably from the vampire bat *Desmodus rotundus.*

Adenoviruses primarily spread through the fecal–oral route. In such cases, infection occurs when susceptible individuals come into direct contact with feces or contaminated objects containing viable viral particles. Respiratory transmission is also possible, occurring when the virus contacts the ocular and nasal conjunctivae [18]. It is noted that *Leopardus pardalis*, among other mammals, uses latrines, which are sites where feces and urine are frequently deposited. These latrines serve as important sites for communication and social interaction among individuals [55,56,57]. In addition, other animals have been recorded using and interacting with *Leopardus pardalis* latrines. King and colleagues [58] documented at least 14 mammal species visiting *Leopardus pardalis* latrines in Costa Rica. These species interacted with the latrines by rubbing their bodies and faces on the sites, sniffing the area, and possibly ingesting material deposited there [58]. In the RNV, latrines of this feline were visited by 19 mammal species, including individuals from the families Felidae, Procyonidae, Mustelidae, Tayassuidae, Cervidae, Tapiridae, Cuniculidae, Dasyproctidae, Caviidae, Sciuridae, Dasypodidae, Myrmecophagidae, Didelphidae, and Leporidae [57]. Human-derived Adenoviruses are capable of surviving for extended periods depending on environmental conditions [59]. Thus, the persistence of Adenovirus particles in the feces of *Leopardus pardalis* could enable this wild feline to disseminate the viral particles in the environment and potentially transmit them to new hosts, after the contact with or the ingestion of feces, assuming that the viral particles remain viable and infectious. This would make latrines important sites for viral transmission to other mammal species, particularly those that directly interact with the material deposited by the feline. The same applies to other carnivores that use latrines, including *Panthera onca*.

Even though the viral findings may be related to the prey consumed by the felines, the potential risk to these predators from this contact should not be neglected. Although felines appear to be resistant to microorganisms present in their prey, in situations of environmental stress or nutritional deficiencies—both of which can affect immune response—predators could become susceptible to pathogenic agents associated with the ingested prey species [60]. Additionally, it should be considered that Adenoviruses in wild felines have shown the potential to cause systemic infections [18]. These conditions could pose new risks to the populations of these predators and consequently to the ecological balance of the environments. Potential viral spillover events also raise concerns about the emergence of new diseases in domestic animals and humans. This applies to regions where the interaction between wild animals, domestic animals, and humans is frequent due to the presence of wildlife in anthropogenic landscapes or the presence of domestic animals and humans (hunters, for example) inside natural areas. In this context, it becomes evident that the health of ecosystems and its impact on animal health are also risk factors for human health, highlighting the necessity of maintaining the ecological balance in natural environments as part of public health measures in a One Health approach [61,62].

Finally, we highlight some limitations of this study, such as the limited number of fecal samples from which PCR could be performed and the subsequent sequencing of the amplified products, which were restricted to two genes (DNA polymerase and IVa2). Additionally, the lack of viral isolation techniques jeopardized the analysis of the integrity, viability, and infectivity of Adenoviruses present in the feces of wild felines. Therefore, we suggest that further studies be conducted in this area, as well as in other regions, aiming to employ Whole Genome Sequencing (WGS) techniques for viral particles’ characterization and other methods capable of assessing the infectivity of Adenoviruses in the fecal samples of felines.

This study provides new insights into the presence of *Mastadenovirus* DNA in fecal samples of *Panthera onca* and *Leopardus pardalis*, emphasizing the possibility of these predators acting as potential viral dispersing agents, and thereby helping to understand the ecological dynamics of these viruses in natural environments. The findings underscore the importance and applicability of these feline species as sentinel animals, serving as tools for investigating the health of the ecosystem and other species, and playing a significant role in viral surveillance programs.

## Figures and Tables

**Table 1:** Amino acid percent identities of the partial DNA polymerase gene comparing obtained sequences with GenBank sequences showing >85% amino acid identity. Analyses were conducted using MEGA7.

Identification	VS631	*Desmodus* Mastadenovirus/PGT-0382	*Desmodus* Mastadenovirus/PGT-03480	VS649	VS652	VS655	*Mastadenovirus bosprimum*	*Caprelous mastadenovirus*
VS631								
*Desmodus rotundus* mastadenovirus/PGT-0382	87							
*Desmodus rotundus* mastadenovirus/PGT-0348	87	100						
VS649	61.2	68.5	68.5					
VS652	57.4	63.1	63.1	94.7				
VS655	59.3	74.7	74.7	94.7	92.6			
*Mastadenovirus bosprimum*	59.3	76.8	77	94.7	92.6	100		
*Caprelous mastadenovirus*	59.3	77.8	78	77.2	77.9	88.2	85.3	

*Desmodus rotundus* mastadenovirus (AOS88389 and AOS88390); *Mastadenovirus bosprimum* (YP_094032); *Caprelous mastadenovirus* (DBA50533); VS631 (PQ347814); VS649 (PQ347815); VS652 (PQ347816); VS655 (PQ347817).

**Table 2:** Amino acid identities (%) of the partial IVa2 gene comparing the obtained sequence with sequences available in GenBank showing the highest identities. Analyses were conducted using MEGA7.

Identification	VS631 IVa2	*Desmodus* sp. Mastadenovirus	*Nyctalus noctula* Mastadenovirus/119/08	*Nyctalus noctula* Mastadenovirus/150/08	*Myotis myotis* Mastadenovirus	*Pipistrellus* sp.
VS631 IVa2						
*Desmodus* sp. mastadenovirus	68.57					
*Nyctalus noctula* mastadenovirus/119/08	51.43	73.81				
*Nyctalus noctula* mastadenovirus/150/08	51.43	73.81	97.62			
*Myotis myotis* mastadenovirus	51.43	76.19	83.33	84.52		
*Pipistrellus* sp.	51.43	74.64	82.14	83.33	82.14	

*Desmodus* sp. mastadenovirus (DBA51399); *Nyctalus noctula* mastadenovirus (AIL52700 and AIL52701); *Myotis myotis* mastadenovirus (AIL52689); *Pipistrellus* sp. (WXG22480); VS631 IVa2 (PQ347813).

## Data Availability

The sequences are deposited in GenBank (accession numbers PQ347813 to PQ347817).

## Supplementary Materials

The following supporting information can be downloaded at: https://www.mdpi.com/article/10.3390/vetsci11100511/s1, Table S1: Adenovirus DNA polymerase sequences included in the phylogenetic analyses.
