# Supplementary material for: Occurrence of Adenovirus in Fecal Samples of Wild Felids (Panthera onca and Leopardus pardalis) from Brazil: Predators as Dispersing Agents?
Source: Vet Sci. 2024 Oct 17;11(10):511. doi: 10.3390/vetsci11100511 (PMC11512381; doi:10.3390/vetsci11100511)
Supplement: Supplementary file 1 [file vetsci-11-00511-s001.zip › vetsci-3241329-Table S1.pdf]

	DNAPOL SEQUENCES
1	AOS88389.1_DNA_polymerase_partial_Desmodus_rotundus_Adenovirus_1
2	AOS88390.1_DNA_polymerase_partial_Desmodus_rotundus_Adenovirus_1
3	AGG81655.1_DNA_polymerase_protein_partial_Desmodus_rotundus_adenovirus_POA/2012/N5.1
4	WMV64464.1_DNA_polymerase_partial_Bat_mastadenovirus_desmodus
5	AOS88399.1_DNA_polymerase_partial_Desmodus_rotundus_Adenovirus_2
6	DBA51400.1_MAG_TPA_asm:_AdPolB_Adenovirus_desmodus35011
7	AOS88398.1_DNA_polymerase_partial_Desmodus_rotundus_Adenovirus_2
8	YP_094032.1_pol_Bovine_mastadenovirus_A
9	DBA50533.1_MAG_TPA_asm:_AdPolB_Adenovirus_capreolus32301
10	BCT90766.1_DNA_polymerase_Bovine_adenovirus_2
11	AP_000006.1_pol_Ovine_mastadenovirus_A
12	AP_000236.1_pol_Porcine_mastadenovirus_C
13	WKY20678.1_DNA_polymerase_partial_Bat_mastadenovirus
14	WKY20683.1_DNA_polymerase_partial_Bat_mastadenovirus
15	QID58106.1_DNA_polymerase_partial_Adenovirus_PREDICT_AdV-13
16	PP259354.1_Feline_adenovirus_strain_hungary
17	AAB38716.1_DNA_polymerase_Canine_adenovirus
18	AAB53750.1_DNA_polymerase_partial_Murine_adenovirus_1
19	AAD09719.1_DNA_polymerase_Bovine_adenovirus_3
20	ABB17778.1_E2B_DNA_polymerase_Human_adenovirus_B3
21	ACZ92148.2_DNA_polymerase_Human_adenovirus_18
22	ADZ39836.1_DNA_polymerase_Simian_adenovirus_49
23	AEW43611.1_polymerase_Chimpanzee_adenovirus_Y25
24	AFD10561.1_DNA_polymerase_Simian_adenovirus_18
25	AGK27095.1_pol_baboon_adenovirus_1
26	AGK27165.1_pol_Simian_mastadenovirus_C
27	AIT70973.1_DNA_polymerase_Simian_adenovirus_DM-2014
28	ALE30388.1_pol_Simian_adenovirus_16
29	ALE30424.1_pol_Simian_adenovirus_19
30	ALE30460.1_pol_Simian_adenovirus_17
31	AQQ73625.1_polymerase_Harbour_porpoise_adenovirus_1
32	ART33363.1_DNA_polymerase_Deer_mastadenovirus_B
33	AXE75624.1_DNA_polymerase_Rousettus_aegyptiacus_adenovirus
34	BAH18775.1_polymerase_Human_adenovirus_D8
35	BBE29304.1_DNA_polymerase_Bat_mastadenovirus
36	BBE29334.1_DNA_polymerase_Bat_mastadenovirus_A
37	BBF72826.1_DNA_polymerase_Eidolon_helvum_adenovirus
38	CAA51882.1_DNA_polymerase_Human_adenovirus_12
39	NC_030874.1_Bat_mastadenovirus_WIV13_complete_genome
40	NP_040516.2_DNA_polymerase_Human_mastadenovirus_C
41	NP_040853.1_DNA_polymerase_Human_mastadenovirus_F
42	NP_064286.1_DNA_polymerase_Bovine_adenovirus_2
43	NP_758820.1_DNA_polymerase_Tree_shrew_adenovirus_1
44	QDA77076.1_DNA_polymerase_Bat_mastadenovirus_B
45	QIZ64149.1_DNA_polymerase_Guinea_pig_adenovirus_1
46	SMG83440.1_pol_Bottlenose_dolphin_adenovirus_1
47	UDF05958.1_DNA-dependent_DNA_polymerase_reindeer_adenovirus_1
48	ULR16484.1_DNA_polymerase_Bat_mastadenovirus
49	UZF96918.1_DNA-dependent_DNA_polymerase_Bovine_adenovirus_10
50	WGN96456.1_DNA_polymerase_protein_Lemur_mastadenovirus

51	YP_067908.1_pol_protein_Simian_adenovirus_3
52	YP_068023.1_pol_Human_mastadenovirus_E
53	YP_213966.1_pol_simian_adenovirus_1
54	YP_001974426.1_DNA_polymerase_Human_mastadenovirus_D
55	YP_002822207.1_DNA_polymerase_Murine_adenovirus_3
56	YP_004782100.1_DNA_polymerase_Bat_adenovirus_2
57	YP_005271182.2_DNA_polymerase_bat_adenovirus_3
58	YP_007518070.1_DNA_polymerase_Simian_adenovirus_20
59	YP_007905999.1_pol_Baboon_adenovirus_3
60	YP_009162345.1_DNA_polymerase_Equine_adenovirus_2
61	YP_009162588.1_pol_Skunk_adenovirus_PB1
62	YP_009174009.1_pol_Simian_adenovirus_13
63	YP_009174940.1_pol_Simian_adenovirus_8
64	YP_009246347.1_DNA_polymerase_Bat_mastadenovirus_WIV9
65	YP_009246380.1_DNA_polymerase_Bat_mastadenovirus_WIV10
66	YP_009246419.1_DNA_polymerase_Bat_mastadenovirus_WIV11
67	YP_009272542.1_DNA_polymerase_Equine_adenovirus_1
68	YP_009272875.1_DNA_polymerase_Bat_mastadenovirus_WIV12
69	YP_009325336.1_pol_Bat_mastadenovirus_G
70	YP_009328902.1_pol_simian_adenovirus_55
71	YP_009362839.1_DNA_polymerase_Bat_mastadenovirus_WIV17
72	YP_009388311.1_DNA_polymerase_Bat_mastadenovirus_WIV18
73	YP_009389490.1_DNApol_red_squirrel_adenovirus_1
74	YP_009505686.1_DNA-dependent_DNA_polymerase_Bottlenose_dolphin_adenovirus_1
75	YP_009553556.1_DNA_polymerase_Polar_bear_adenovirus_1
76	YP_010087257.1_DNA-dependent_DNA_polymerase_Ovine_adenovirus_8
77	YP_010796138.1_pol_Porcine_mastadenovirus_B
78	NP_044702.1_DNA_polymerase_Duck_atadenovirus_A
79	NP_077389.1_pol_Bovine_atadenovirus_D
80	NP_659515.1_pol_Ovine_ataadenovirus_7
81	YP_009414575.1_DNA_pol_Odocoileus_atadenovirus_1
82	YP_001552247.1_DNA_polymerase_Snake_ataadenovirus_1
83	YP_009051654.1_DNA_polymerase_Lizard_atadenovirus_2
84	YP_009047094.1_DNA_polymerase_Pigeon_aviadenovirus_1
85	YP_008719852.1_DNA_polymerase_Turkey_aviadenovirus_5
86	YP_008719820.1_DNA_polymerase_Turkey_aviadenovirus_4
87	YP_006383556.1_DNA_polymerase_Goose_aviadenovirus_4
88	YP_004346921.1_DNA_polymerase_Fowl_aviadenovirus_C
89	YP_004191813.1_DNA_polymerase_Fowl_aviadenovirus_E
90	QKW89978.1_DNA_polymerase_Duck_aviadenovirus_B
91	QJB76054.1_DNA_polymerase_protein_Fowl_aviadenovirus_D
92	NP_050281.1_DNA_polymerase_Fowl_aviadenovirus_D
93	YP_009310429.1_DNA_polymerase_Pigeon_aviadenovirus_2
94	AP_000478.1_pol_Turkey_siadenovirus_A
95	NP_062435.1_pol_Frog_siadenovirus_1
96	YP_004414800.1_DNA_polymerase_Raptor_siadenovirus_1
97	YP_009252208.1_DNA_polymerase_Chinstrap_penguin_siadenovirus_2
98	AFP53934.2_DNA-dependent_DNA_polymerase_Red-eared_slider_adenovirus_1
99	QCQ84150.1_DNA-dependent_DNA_polymerase_White_sturgeon_adenovirus_1

	IVa2 SEQUENCES
1	DBA51399.1_MAG_TPA_asm:_PackIVa2_Adenovirus_desmodus35011
2	AIL52700.1_IVa2_partial_common_noctule_adenovirus_2
3	AIL52701.1_IVa2_partial_common_noctule_adenovirus_3
4	AIL52689.1_IVa2_partial_greater_mouse-eared_bat_adenovirus_1
5	WXG22480.1_MAG:_pIVa2_protein_Bat_mastadenovirus_blast
6	YP_004414799.1_IVa2_Raptor_siadenovirus_1
7	YP_009252207.1_IVa2_Chinstrap_penguin_siadenovirus_2
8	AP_000477.1_IVa2_Turkey_siadenovirus_A
9	YP_006383555.1_encapsidation_protein_IVa2_Goose_aviadenovirus_4
10	QKW89977.1_pIVa2_Duck_aviadenovirus_B
11	YP_008719851.1_IVa2_turkey_aviadenovirus_5
12	YP_004346920.1_IVa2_Fowl_aviadenovirus_C
13	YP_008719819.1_IVa2_turkey_aviadenovirus_4
14	YP_004191812.1_pIVa2_Fowl_aviadenovirus_E
15	NP_050280.1_maturation_protein_Fowl_aviadenovirus_D
16	QJB76053.1_encapsidation_protein_Fowl_aviadenovirus_D
17	YP_009310428.1_encapsidation_protein_IVa2_Pigeon_aviadenovirus_2
18	YP_009047093.1_encapsidation_protein_IVa2_Pigeon_aviadenovirus_1
19	QCQ84149.1_IVa2_White_sturgeon_icthadenovirus_1
20	YP_009051653.1_protein_IVa2_Lizard_atadenovirus_2
21	YP_001552246.1_IVa2_Snake_atadenovirus_1
22	YP_009414574.1_IVa2_protein_Odocoileus_atadenovirus_1
23	NP_659514.1_IVa2_(37.5kd) Ovine_atadenovirus_7
24	NP_077388.1_protein_IVa2_Bovine_atadenovirus_D
25	BBF72825.1_IVa2_Eidolon_helvum_adenovirus
26	YP_009362838.1_IVa2_Bat_mastadenovirus_WIV17
27	YP_009388310.1_IVa2_Bat_mastadenovirus_WIV18
28	AXE75623.1_IVa2_Egyptian_fruit_bat_adenovirus
29	YP_009272874.1_IVa2_Bat_mastadenovirus_WIV12
30	YP_009272921.1_IVa2_Bat_mastadenovirus_WIV13
31	UZF96917.1_IVa2_Bovine_adenovirus_10
32	YP_010087256.1_IVa2_Ovine_adenovirus_8
33	AAD09718.1_IVa2_Bovine_adenovirus_3
34	ART33362.1_IVa2_Deer_mastadenovirus_B
35	UDF05957.1_IVa2_protein_reindeer_adenovirus_1
36	YP_009162344.1_IVa2_Equine_adenovirus_2
37	YP_009174008.1_IVa2_Simian_adenovirus_13
38	AIT70972.1_IVa2_Simian_adenovirus_DM-2014
39	ACZ92147.1_IVa2_protein_Human_adenovirus_18
40	CAA51881.1_maturation_protein_(protein_IVA2)
41	ALE30387.1_IVa2_Simian_adenovirus_16
42	YP_009174939.1_IVa2_Simian_adenovirus_8
43	ADZ39835.1_IVa2_Simian_adenovirus_49
44	AGK27094.1_IVa2_baboon_adenovirus_1
45	YP_213965.1_IVa2_simian_adenovirus_1

46	AGK27164.1_IVa2_Simian_mastadenovirus_C
47	YP_007905998.1_IVa2_Baboon_adenovirus_3
48	ALE30423.1_IVa2_Simian_adenovirus_19
49	NP_040852.1_encapsidation_protein_IVa2
50	AFD10560.1_IVa2_Simian_adenovirus_18
51	ALE30459.1_IVa2_Simian_adenovirus_17
52	AP_000201.1_IVa2_Human_adenovirus_5
53	NP_040515.1_encapsidation_protein_IVa2
54	AAQ10542.2_Human_adenovirus_1
55	BAH18774.1_pIVa2_protein_Human_adenovirus_D8
56	YP_001974425.1_encapsidation_protein_IVa2_Human_mastadenovirus_D
57	AEW43610.1_IVa2_Chimpanzee_adenovirus_Y25
58	YP_068022.1_IVa2_Human_mastadenovirus_E
59	AAW33114.1_IVa2_protein_Human_adenovirus_14
60	ABB17777.1_protein_IVa2_Human_adenovirus_B3
61	YP_009328901.1_IVa2_simian_adenovirus_55
62	YP_067907.1_IVa2_Simian_adenovirus_3
63	YP_007518069.1_IVa2_Simian_adenovirus_20
64	ADR77833.1_IVa2_Murine_adenovirus_2
65	YP_009162587.1_IVa2_protein_Skunk_adenovirus_PB1
66	BBE29303.1_IVa2_Bat Mastadenovirus
67	QDA77075.1_IVa2_Bat_mastadenovirus
68	YP_004782099.1_IVa2_Bat mastadenovirus
69	YP_009272541.1_maturation_protein_Equine_adenovirus_1
70	AAB38715.1_IVa2_protein_Canine_adenovirus_2
71	YP_009325335.1_IVa2_Bat_mastadenovirus_G
72	BBE29333.1_IVa2_Bat_mastadenovirus_A
73	YP_005271181.1_IVa2_protein_bat_adenovirus_3
74	QIZ64148.1_IVa2_Guinea_pig_adenovirus_1
75	YP_002822206.1_pIVa2_Murine_adenovirus_3
76	YP_010796136.1_IVa2_Porcine_mastadenovirus_B
77	YP_009200.1_IVa2_Porcine_adenovirus_3
78	SMG83439.1_IVa2_Bottlenose_dolphin_adenovirus_1
79	YP_009389489.1_IVa2_red_squirrel_adenovirus_1
80	YP_009553555.1_IVa2_Polar_bear_adenovirus_1
81	WGN96455.1_IVa2_protein_Lemur_mastadenovirus
82	ULR16483.1_IVa2_Bat mastadenovirus C
83	YP_009246346.1_IVa2_Bat_mastadenovirus_WIV9
84	YP_009246418.1_IVa2_Bat_mastadenovirus_WIV11
85	YP_009246379.1_IVa2_Bat_mastadenovirus_WIV10
86	AP_000005.1_IVa2_Ovine_mastadenovirus_A
87	YP_094031.1_IVa2_Bovine_mastadenovirus_A
88	AP_000235.1_IVa2_Porcine_mastadenovirus_C
89	YP_009505685.1_pIVa2_Bottlenose_dolphin_adenovirus_1
90	AQQ73624.1_IVa2_Harbour_porpoise_adenovirus_1
